# Compound Fracture of Leg

**Published:** 1893-01

**Authors:** T. D. Stow

**Affiliations:** Mexico, N. Y.


### COMPOUND FRACTURE OF LEG.

T. D. Stow, M. D., Mexico, N. Y.

On the evening of February 10th, 1892, was called to see F. L. K., a tall, heavy young man of twenty years, who sustained a fracture of the tibia and fibula in the lower third. There were two wounds on the inner face of leg communicating with the broken ends of bone, from which there was not rapid, but continued flow of blood. The leg—right one—was then not badly swollen, nor was there much ecchymosis. He was a stone-cutter, and was at work on a shaft of granite, that rested on one of its sides some three feet from the floor, the shaft weighing some nine hundred pounds. As he was trying to move the blocks that supported the shaft, one of them slipped, and the shaft of granite fell to the floor, breaking his leg, as before stated. The wonder is, his leg was not crushed to a jelly. The accident occurred in a neighboring town, and he was brought home on a railway train. I did not examine the limb until the next day, for it had been dressed by two physicians living in the village of Sandy Creek, where he was hurt. The limb rested as it was at first placed, on a double-inclined Day-splint, for some ten days. After that, I put it in a newly-designed fracture box, having a half-circle copper trough in the bottom, with conducting-pipe and rubber-tubing attached, by which to convey pus and irrigating fluid into a pail below. For some five days from the date of accident there was little change in the color of the leg, though the swelling increased, the reactive power seeming to be "knocked out." Suddenly, the condition changed. High inflammation arose in the whole limb—particularly in calf of leg and the parts nearest the line of fracture—which was oblique; great swelling and discoloration of the limb, pain, beating, and great soreness of the limb, with dread of having it touched.

At the time I put the leg in the fracture-box, rubber drainage-tubes were inserted, and from this flowed a copious discharge of sanious matter. This discharge became so offensive and so profuse that it seemed as if the limb would be destroyed entirely. Of course, this high-graded inflammation delayed union of the parts, so that on the twenty-fifth day of his confinement we anæsthetized him, opened the mouth of wound, and explored the cavity. Some small spiculæ of bone were removed; a pocket was found burrowing among the sural muscles; a sinus had formed beneath the tibia, one branch opening midway between the tibia and fibula, another ran over the crest-spine of tibia—communicating with the posterior sinus and formed a pocket, having dissected off the tibial periosteum at upper end of the lower fragment. This revelation of the traumatic condition called for some means of drainage, not yet adequate to the gravity of the case. So, making a new opening through the sural muscular tissues,I passed a three-eighths Para rubber tube through the entire limb, from the uppermost pocket between the tibia and fibula, thus directing the discharges of purulent matter and the hot-water irrigation nearly perpendicular downwards, into the conducting trough and thence into the catch-pail.

The upper end of tube was kept corked to prevent the access of air and of dirt and the whole lower half of leg was daily packed in pure absorbent cotton; the rest of the leg, including the parts contiguous to drainage-tube, was covered with butchers' linen that was left long at the ends above for facilitating the raising and adjustment of limb in case of necessity. The leg above and below the copper trough at the bottom of box was packed in fine, charred sawdust. The sides of box were hinged two and a half inches up from the bottom, so that they could be let down to examine the leg and clear out the soiled sawdust and put in clean when necessary. The thigh and leg below the knee were kept in place by straps that, passing through the inside of box, outside the bottom, and then securely buckled over pads on the anterior face of limb. Counter-extension was effected by a strap of inch-wide webbing passed through mortices in foot-board, and tied on the outside. Much difficulty was experienced in nicely adjusting this on account of the uneasiness of the patient, the swelling of foot, and burning in heel. Normal posture of leg and foot were secured at will by peculiar packing of the sawdust and lateral rotation of foot by means of broad tape, or packing the foot at sides with cotton. A board some three feet long and ten inches wide was placed upon the bed under the limb to form a track. The fracture-box, resting on two rollers, was placed on this, so that any movement of patient upward or downward should cause the least displacement of fragments. After the operation of March 4th, the twenty-fifth day after the injury, the patient began to improve and union of the fragments commenced. From the 15th of February until the 25th of March each day seemed to prognosticate amputation, and if I had listened to and followed the advice and fears expressed by his neighbors his leg would be off to-day. So far I have perhaps tediously detailed the mechanism of the injury and of the chirurgical work; but this is but part of the story. There were certain unpleasant and grave

### Complications.

1st.—He was a great smoker and was getting into the habit of guzzling beer and whiskey, and was brought home considerably intoxicated at the time of injury.

2d.—Following an attack of grippe of the early spring of 1891, he was treated "*secundum artem*," quinined, etc., he had a bronchial inflammation, with cough, etc.

3d.—Between February 12th and 26th, he had an attack of mucous dysentery—enteritis.

4th.—Burning in calf of leg and heel were a source of great annoyance. The patient often felt obliged to tinker at the position of the leg and heel, moving the packing and re-adjusting the counter-extension, much to his injury and my vexation and disgust.

### The Homœopathics of the Case.

It is always homœopathic, in the treatment of fractures, to adjust the broken parts, for that is the placing of the broken parts in a condition similar to the natural or sound one, but surgical conditions and homœopathic therapeutics are still more and better related. The subject of this sketch was medicated as follows:

1st.—For five days he had Arnica$^{30c}$, in water, three doses a day to cover: The character of the injury, the suggillation of soft parts, the pain and soreness and his dread of motion and of having the parts handled or touched, dry hacking cough worse at night, harassing cough with expectoration of small somewhat spherical masses containing spots of blood, soreness felt inside the chest, stitches like pleurisy in left side, etc.

2d.—For frequent, small, slimy, blood-streaked stools, with straining, tenesmus, chilliness, pain and urging before stool, nausea, foul breath and dirty, yellow, flabby tongue he had Mercurius-sol$^{cc}$. Rápid improvement followed.

3d.—The burning in heel and in calf of leg were often relieved by Arg-mur. and Graph.

He was confined to the bed some eleven weeks, the limb being kept in the fracture-box fifty-six days. On the day his limb was taken out of the fracture-box a plaster-of-Paris bandage was applied from the base of toes to the knee, this was worn some four weeks. There was good union of the fragments; little or no shortening, and the limb straight. A little tilting inward of the upper end of lower fragment has prevented the entire healing of the uppermost wound, and there is a slight sanious discharge with a little pus at times. He lost forty pounds during his sickness, but has nearly recovered that, and he is now able to walk with a cane. If he eschews tobacco and liquor his prospects will brighten from time to time until he gets full use of his limb. He was in the office on the 8th, and I gave him Sil.$^{cc}$. We shall see how it acts.

